# Methodological considerations in interpreting a process evaluation of antimicrobial stewardship for community-acquired pneumonia

**DOI:** 10.1017/ash.2026.10397

**Published:** 2026-05-20

**Authors:** Rhushvi Thakkar, Deepika Raina, Chandana Maji, Vaseem Anjum

**Affiliations:** 1 Dr. D. Y. Patil Medical College, Hospital and Research Centre, Dr. D. Y. Patil Vidyapeeth (Deemed-to-be-University), India; 2 Faculty of Pharmaceutical Sciences, Graphic Era Hill University, India; 3 Centre for Promotion of Research, Graphic Era Deemed University, India; 4 NIET: Noida Institute of Engineering and Technology, India; 5 Malla Reddy Institute of Medical Sciences, India

Dear Editor,

The recently published process evaluation of a multicomponent antimicrobial stewardship intervention for hospitalized community-acquired pneumonia usefully complements the parent stepped-wedge cluster-randomized trial.^1^ Its strengths include embedding process inquiry within a pragmatic trial, combining quantitative exposure data with qualitative interviews, and purposively sampling hospitals with differing performance. These design features improve contextual understanding of implementation across sites and are well aligned with current thinking on evaluation of complex interventions.^2,3^.

However, some conclusions appear stronger than the methods can fully support. The article states an aim to assess the relative contribution of intervention components, yet exposure was not measured for several components, including pocket cards, posters, and opinion leaders, and the qualitative sample comprised only 11 participants from 4 of 9 hospitals, interviewed 2 years after the trial end point. For multicomponent interventions, current methodological guidance emphasizes explicit linkage between context, mechanisms, fidelity, and outcomes. Without fuller component-level measurement and a prespecified plan for triangulating process data with trial outcomes, judgments about which components were “most effective” are better interpreted as perceptions of salience than as causal attribution.^2,3^

Interpretation of between-hospital heterogeneity also warrants caution. In stepped-wedge cluster-randomized trials, intervention effects are estimated through both within-cluster and between-cluster comparisons over time, with adjustment for secular trends. Accordingly, post hoc hospital-level explanations based largely on retrospective interviews should be viewed as hypothesis-generating rather than confirmatory. In addition, the predominance of local opinion leaders among interviewees may have introduced recall and social desirability bias toward components that were most visible or professionally endorsed.^4^

These considerations do not diminish the study’s practical value. Rather, they suggest that the findings are most persuasive as an implementation-focused description of feasible stewardship strategies, not as a definitive ranking of component effectiveness. Future process evaluations in this field would be strengthened by prospective measurement of fidelity, dose, reach, and context for every component, together with an a priori strategy to integrate qualitative insights with trial outcomes.

## Data Availability

Not applicable.

## CRediT author statement

**Rhushvi Thakkar:** Conceptualization; Writing—Original Draft; and Writing—Review and Editing. **Deepika Raina:** Conceptualization; Writing—Original Draft, and Writing—Review and Editing. **Chandana Maji**: Writing—Original Draft and Writing—Review and Editing. **Vaseem Anjum:** Validation and Writing—Review and Editing.
